# What to do when patients and physicians disagree? Qualitative research among physicians with different working experiences

**DOI:** 10.1007/s41999-020-00312-3

**Published:** 2020-04-02

**Authors:** Rozemarijn Lidewij van Bruchem-Visser, Inez Duconia de Beaufort, Francesco Umberto Salvatore Mattace-Raso, Ernst Johan Kuipers

**Affiliations:** 1 Department of Internal Medicine, Erasmus MC University Medical Center, Dr. Molewaterplein 40, 3015GD Rotterdam, The Netherlands; 2 Medical Ethics and Philosophy of Medicine, Erasmus MC University Medical Center, Rotterdam, The Netherlands

**Keywords:** Distress, General medicine, Terminal care, Treatments

## Abstract

**Purpose:**

Impasses between patients, relatives and physicians occur frequently. With the growing attention for shared decision making, it is valuable to know how impasses arise. To understand the challenges experienced by physicians when their opinion on medical decisions differ from those of patients or relatives.

**Methods:**

Fifteen physicians with different working experiences, from five medical specialties were interviewed using a narrative approach. Interviews were based on two patient stories provided by the physician. First of a patient (or relative) who did not want to adhere to a treatment the physician deemed necessary, and the second of a patient (or relative) who requested a treatment the physician felt was unnecessary. Data were analyzed using a bottom-up approach, with identification of five themes (autonomy of the patient, communication, emotions, circumstances and metaphors). Twenty subthemes were formed.

**Results:**

693 references were made. Six major nodes were identified: frustration experienced by the physician, role of the relatives, agreement, cultural/religious aspects, comprehension by the patient of the situation and the existence of an established relationship between patient and physician.

**Conclusions:**

Physicians felt uncomfortable when there was disagreement between themselves and patients or relatives. Frustration was felt when relatives spoke on behalf of the patient, while there was no evidence the desired decision was ever expressed by the patient. A disagreement with a patient was described as being less frustrating, when the patient was able to explain the reasons for making a decision. Differences in background, especially religious, were often mentioned as complicating communication.

## Key summary points

**Aim:**

To understand the challenges experienced by physicians when their opinion on medical decisions differ from those of patients or relatives.

**Findings:**

Physicians felt uncomfortable when there was disagreement between themselves and patients or relatives. Frustration was felt when relatives spoke on behalf of the patient, while there was no evidence the desired decision was ever expressed by the patient. A disagreement with a patient was described as being less frustrating, when the patient was able to explain the reasons for making a decision. Differences in background, especially religious, were often mentioned as complicating communication.

**Message:**

Efforts must be made to establish a bond of trust between patient, relatives and physician. The use of advance directives should be encouraged. In case of an impasse between a physician and patient or relative, advice can be sought from other professionals.

## Introduction

In recent years, much effort was invested in implementing shared decision making (SDM). In this process, patients and health professionals together decide on clinical approaches, discussing all information, risks and benefits as well as patient values and preferences. The primary reason to promote SDM is to respect the autonomy of the patient, being the subject of treatment. Another reason is that engaged, informed patients are more satisfied with the chosen treatment [1]. From the point of view of the physician, the goal is to decide on treatment options in a dialog with the patient, considering guidelines and with respect for his or her professional judgment.

As far back as the mid-1970s, attempts have been made to reduce medical overuse. Nowadays, this struggle continues. Expectations and demands of patients can result in clinicians feeling pressured to provide low-value care. Furthermore, physicians tend to opt more easily to start a treatment, as opposed to withholding treatment. Initiatives such as the Choosing Wisely campaigns seek to advance a dialog on avoiding unnecessary medical tests, treatments and procedures [2]. The goal of these initiatives is to improve quality of care. One of the most important findings in the International Health Policy (IHP) survey was that 57% of Dutch general practitioners (GPs) thought patients received too much health care [3]. A recent survey in the USA in over 2000 physicians revealed the belief among physicians was that 20.6% of overall medical care was unnecessary [4]. Reducing overuse also provides economic benefit. Previous studies reported that at least 20% of health-care spending in the USA was unnecessary [5].

This spiral of events has been described as a “Perfect Health Storm” [6]. Four physician-related factors driving overuse have been identified: physician culture, fee-for-service payment, marketing and the fear of being sued for medical malpractice [6]. Dutch investigators described 15 mechanisms that can lead to excessive and excessively prolonged treatment [7]. Besides suggested treatments, patients or relatives can also request other treatments, based on their own beliefs. Problems arise if in the professional judgment of the physician, the preferred treatment is unacceptable.

We interviewed physicians to gain insight into how they experienced an impasse between themselves and a patient concerning treatment options. The study goal was to understand the challenges experienced by physicians when their opinion on medical decisions differs from those of patients or their relatives.

## Methods

We interviewed physicians using a narrative approach. This allows participants to share their subjective experiences and by doing so also reinterpret and give further meaning to these experiences [8, 9]. We used this to do an in-depth exploration of how physicians experienced a difference of opinion with a patient or relatives regarding medical decisions. This method particularly suited this study as so far little is known about physicians’ subjective experiences when being faced with patients with a different opinion about their clinical management.

### Recruitment of participants

We purposively invited 18 physicians to participate. Invitees were working in the south-west of the Netherlands with different years of working experience. Fifteen physicians, from five different medical area’s (internal medicine, general practice (GP), intensive care, surgery, and oncology), agreed to participate. Their work experience ranged from 1 to 35 years. All interviews were conducted face to face. Before the interview, the participants were informed that the interview would deal with “disagreement between physician and patient”. Participants were asked to think of two different cases from their experience: one in which the patient did not want to receive treatment, whereas the physician judged it necessary; and one in which the patient wanted prolonged or more intense treatment, while the physician considered this unnecessary or harmful. No limitations were given regarding type of underlying disease, patients’ age, sex or cultural background. Participants were given written and verbal information and gave informed consent. Ethical approval was granted by the ethical committee of the Erasmus MC.

### Data collection and analysis

Interviews were digitally recorded and transcribed verbatim with the interviewees’ permission. The interviewer guides were flexible, allowing prompt and open questions to encourage participants to talk in depth about their experiences and perceptions. Basic demographic information of the patients (age, sex, ethnicity and religion was recorded (see Table 1). It was also noted whether or not there was an existing patient–physician relationship prior to the described situation) and whether or not an impasse between patient (or relatives) and the physician was described by the physician. Basic information of the physicians (sex, medical specialty, years of working experience) was also recorded (see Table 2). The study was facilitated by QSR NVivo 12 software (QRS International Pty Ltd, Melbourne, Victoria, Australia).

**Table 1:** Descriptive characteristics of patients

	Physician wants to continue	Physician wants to stop
Total no of patients	15	14
Aged 25–45	2	6
Aged 46–65	4	2
Aged 65 years or older	9	5
Age not described		1
Male	10	3
Female	5	10
Unknown	0	1
Underlying disease		
Malignancy	5	5
Kidney disease	3	2
Diabetes mellitus	2	0
Neurological condition	2	4
Chronic obstructive pulmonary disease	1	0
Infection	1	0
Complex surgery	1	1
Medically unexplained	0	1
Dementia	0	1
Religious background	1	10
Muslim	1	5
Christian	0	2
Other	0	3
Non-religious/not mentioned	14	4
Existing relationship	11	7
Impasse mentioned	5	10

**Table 2:** Descriptive characteristics of the interviewed physicians

Participant	Sex	Specialty	Working experience (years)
1	Female	General practitioner	22
2	Male	Intensive care	10
3	Female	Oncology	11
4	Male	Surgery	30
5	Female	Intensive care	35
6	Male	Internal medicine	4
7	Female	Oncology	20
8	Female	General practitioner	11
9	Female	Internal medicine	1
10	Female	Internal medicine	15
11	Male	Surgery	1
12	Male	Surgery	10
13	Female	Oncology	2
14	Female	Intensive care	6
15	Female	General practitioner	3

After conducting 15 interviews, data were analyzed to see if new themes were emerging. This was not the case; therefore data saturation was reached.

The transcripts were coded by two independent researchers (RvB and LD) to ensure rigorous analysis. The first five interviews were used to compare coding strategies. No significant differences were found. The first author developed further interpretation of the results with regular comments from the other authors.

We used a bottom-up approach to identify recurring themes. This was performed according to the method specified by Braun and Clarke [8].

1. Transcripts were read and re-read to familiarize the researchers with the data.
2. Systematic line by line coding to identify common features.
3. Codes were reviewed to determine potential themes.
4. Themes were reviewed for internal homogeneity and external heterogeneity to ensure coherence and distinction.
5. Themes were identified and named.

Five themes were identified: patient autonomy, communication, emotions, circumstances and metaphors. Twenty subthemes were formed based on the interviews (see Table 3). In total, 693 different references were identified as matching with at least one (sub)theme.

**Table 3:** Themes and subthemes, number of references and files

Theme	Subtheme	References	Files
Autonomy of the patient		38	18
	Rights	10	9
	Role of relatives	72	22
	Responsibility	33	19
Communication		14	10
	Providing information	51	23
	Listening	14	12
	Agreement	74	28
	Talking about death	22	9
Emotions		13	10
	Fear	15	6
	Anger	8	4
	Hope	10	7
	Frustration by patient	4	4
	Sadness	4	4
	Frustration by physician	37	16
Circumstances		17	14
	Culture/religion	24	11
	Financial matters	9	4
	Comprehension of situation	32	20
	Environment/setting	24	13
	Social network	26	15
	Patients’ disease	61	28
	Relationship with patient	38	28
Metaphors		43	16

## Results

Fourteen physicians described two different patients. One physician only discussed a patient who wanted to continue treatment (interview no. 4).

In 14 out of the 15 patient stories where the physician wanted to continue treatment and the patient wanted to stop, there was direct communication between physician and the patient. Of the 14 cases where the physician wanted to stop treatment, there was direct communication between the patient in three cases. In the other 11 cases the relatives either played a prominent role, accompanying the patient (two cases) or the communication was between physician and relatives only (nine cases).

Six major nodes were identified: frustration experienced by the physician, the role of the relatives, agreement, cultural/religious aspects, comprehension by the patient of the situation and existing of an established relationship between patient and physician. Quotes are identified by participant number.

### Frustration experienced by the physician

In 16 out of 29 cases, physicians spontaneously reported frustration on their part while dealing with patients or relatives. These comments were made both in relation to cases in which patients wanted to continue treatment, as well as those in which the physicians wanted to continue (8 vs. 8 cases). Physicians talked about their frustration and feelings of helplessness when they were unable to convince the patient or relatives of their views.

I have no words to reach her, to relate to her inner world. (1.1)

In the cases where direct communication with the patient was possible, frustration occurred when the physician felt that the patient did not (or was not able to) understand the severity of the condition or the necessity of the proposed treatment. Physicians also expressed frustration when they felt relatives did not recognize them as being persons having emotions of their own.

And nobody pays any attention to the physician. Same as in the case of performing euthanasia, the one crying, it was me. (4.2)

In more than one interview, physicians described their challenges with relatives who demanded more intensive treatment than the physician felt comfortable with. In many of these cases, the patient had never consented to the requested invasive treatments, and this caused physicians’ discomfort. In other cases, the physician considered the requested treatments futile.

I gave her antibiotics, while that was already going against every fiber of my being, but the husband insisted, because that is what his wife would have wanted. And one of the sisters, the one that was most involved, she is a patient of mine, said “should we be giving all this treatments?” or something like that. (8.2)

Remarkably, in the cases where a patient wanted to stop, against advice of the physician, and no frustration was felt, the patient was described as mentally competent and well aware of the options.

### Role of the relatives

Almost all physicians mentioned the role of the relatives. In nine cases, the relatives requested treatments the physician thought would not benefit the patient. In all cases, this concerned a patient who was not able to speak for himself. Several physicians expressed their doubts whether the wish to prolong treatment was prompted by the concern of the relatives for the patient’s well-being or for their own sake.

We will never know what Mrs. X herself thought on the matter. It was entirely the wish of the relatives. (9.2)

The fear of getting blamed for the death of the patient was mentioned, as well as the influence of religion. In 12 interviews, physicians described that they tried to accommodate the requests made by the relatives, until they felt their actions were no longer professionally and humanly justifiable.

For me, that was the turning point, I was continuing (treatment), not for the sake of the patient, but for the sake of the relatives. And when I realized that, I thought: this must stop. This (treatment) should not be for the relatives, it should be about the patient. (5.2)

In some cases, the physicians wanted to continue treatment, but the relatives expressed a desire to stop, often referring to prior expressed wishes by the patient. They expressed a strong wish to honor the request made by the patient, even when both relatives and physician agreed continuation of treatment would be the better option. When a patient expressed a desire not to start a treatment, all physicians complied, be it reluctantly in some cases.

Physicians described relatives wanted to stop treatment because of complications, and they found it difficult to grant that request, as it was unsure what the point of view of the patient actually was. More importantly, the complications were, according to their professional opinion, an unfortunate but foreseeable result of a treatment the patient had consented to. As a result, sometimes the treatment was continued against the wishes of the relatives.

### Agreement

In 28 of the 29 cases, physicians mentioned the topic of (dis)agreement. In most cases, they referred to reaching an agreement, or at least trying to do so, with patient or relatives. This was sometimes a smooth process and in other cases unsuccessful.

And in the end, when he was extubated, we talked to him for quite some time, and as a result we agreed on what limitations he wanted regarding treatments. (14.1)

I had just become an oncologist, and had not yet realized that people could also chose not to start a treatment. So my assumption was that she would want the full chemo, and so I told her what the plans were. And that is when she turned completely furious. (3.1)

When faced with a difficult, sometimes emergency decision, agreement was sought with fellow specialists, and in most cases this helped the physician to decide what to do. Other described strategies were: revisiting the patient or relatives and investing time and effort to build a relationship.

### Cultural/religious aspects

Frequently, the cultural or religious background of the patient and his relatives was mentioned. In the cases of the patient (or relatives) wanting to stop treatment, with the physician advising to continue, there was one comment regarding a cultural background. The physician thought the child of a patient would decide on behalf of the patient, but was surprised the decision was left to the patient. In other cases, the relatives spoke on behalf of the patient, without consulting the patient.

Several physicians described how a difference in background between the patient, relatives and physician caused difficulties in communication.

I think we still make a lot of mistakes, because we are not aware of the way bad news should be addressed in specific cultures. (…) So, in several years, I found out by trial and error that in some cultures it is not customary for younger relatives members, or those lower in the hierarchy, to be allowed to deliver bad news. They are not allowed to say that someone will die (…), or there is an incurable disease. (…) So we only found out after a long time (that the patient was not told the diagnosis). Because the daughter, who had been in the Netherlands for a very long time, spoke fluent Dutch, and was highly educated, understood perfectly well what was happening, but had not told her mother, because she just refused to tell her mother the bad news, the diagnosis that was made.(1.02)

In the description of the patients, physicians often spontaneously emphasized the role of the religious background of a patient or relatives. Of the 14 cases where the physician wanted to stop treatment, in ten cases a religious background was described by the physician. In five cases a Muslim background was described, in two cases a Christian background and in three cases the specific religion was not specified.

This young man stood by his decision, he did not want to receive any blood, and accepted the consequences that that would be the end of it. His parents struggled, but were also Jehovah’s witnesses, so in the end they supported his decision, but it was a real dilemma, he was so young. (3.2)

### Comprehension of the situation

In multiple cases, physicians expressed concerns regarding the level of comprehension of the patient of their situation. Physicians described their efforts to inform their patient in these situations. Multiple visits were made to the patient, help was sought from colleagues and time was invested.

And then we try and talk to them (the relatives) a lot, explain it, show them the scans, as I do with all the patients who suffered a neurological trauma, to make it less abstract, and to explain why we are going to stop the treatment. But, if they have no clue of what is depicted on the scan, that does not help at all. So then we try to explain, what are the different functions of the brain, and what functions are now gone. (5.2)

Sometimes, patients expected the outcome to be worse than the physician anticipated. In those cases, similar efforts were made to convey the (in the words of the physician) “more objective, expected outcome” to the patient.

Often, people do not know, notwithstanding the severity of the situation, everything can turn out just fine. (4.1)

### Established relationship between patient and physician

In the case of an established relationship between the patient and physician, impasses occurred sporadically. Physicians described agreement was reached in multiple conversations, with enough time for all parties to think about the options.

But my supervisor, who had known her for years, said: “she always talks that way when things are not going well”. (9.1)

However, in the case of no previously existing relationship, impasses were more often described.

But well, when you do not know the patient at all, that is a major pitfall (3.1)

Remarkably, an impasse arose in all the cases without an existing relationship in which the patient or relatives requested treatment, whereas the physician wanted to stop. Physicians told about their efforts to get to know the patient, who was in a number of cases unable to communicate. The acuteness of the situation was described as being a major contributor to arising impasses.

## Discussion

In the present study, we found that for the physician the impossibility of direct communication with the patient was frustrating. The frustration seemed to stem from the fact that the right sparring partner, being the patient, was unable to take part in the discussion. If the patient was described as mentally competent and aware of treatment options, physicians still tried to convince them to “see the physicians’ way”, but accepted the divergent desired treatment strategy without frustration.

In several cases, physicians felt uncomfortable with starting the desired treatment, that they set clear boundaries. In almost all cases, here was a line the physician was not willing to cross, to preserve professional and moral standards.

Relatives frequently have to make medical decisions for an incompetent patient acting as surrogate decision maker. Advance directives, in which the patient describes treatment preferences could guide the relatives in this process. In this study, no advance directives were mentioned. This is congruent with studies that show the community prevalence of advance care directives remains low, with percentages ranging between 5 and 20% [11, 12]. So, in most cases, the relatives cannot rely on such a document. Possible problems in communicating with relatives were (among others) identified as the failure to reach a shared view of the patients’ medical condition (and prognosis), problems with applying the principle of substituted judgment, difficulties in addressing the full range of end-of life-decisions and offering the relatives “the wrong choice” [13]. The wrong choice explained as a choice between care and cure, where no cure is possible, as opposed to the choice between prolonging life and quality of life [13].

Although it is to be expected relatives would be adequate in assessing the wishes of the patient, this is not shown by the existing literature. Several studies performed in different categories of patients such as cancer patients, patients with early dementia and dialysis patients, suggest that relatives are often unable to correctly assess the preferences of the patient [14–16]. An interview-based study including 750 patient–caregiver dyads showed that, in case of discordance, patients and caregivers often had an unrealistic optimistic view regarding extent of disease, treatment goals and prognosis [17].

It seems comprehensible that relatives primarily opt for life-prolonging treatment for the patient. In a study on patients on the intensive care, relatives struggled with the conflict between honoring the patients’ wishes and values, and their own emotional problems with “being the one letting a patient die” [18]. We found that agreement was more easily achieved if the patient and relatives were already known by the physician. Establishing a relationship with both patient and relatives in early stages of a disease is important. The triad patient, relatives and physician can prove to be very helpful in case of difficult decisions [19].

In our study, most impasses arose when patients were “new” to the physician as a result of a transfer into a practice or due to an acute presentation in a hospital. Therefore, there was no history between the patient, relatives and physician. Impasses arose especially in the cases in which there was an acute treatment decision to be made (for instance in the emergency department or in the intensive care unit). The acuteness of the situation and the inevitability of the decision are likely to be important factors.

In our study, physicians told the perceived level of understanding the patient (or relatives) seemed to have of the actual situation was an important factor in the arising of an impasse. In a study including interviews of dyads of physicians and patients, unclear expression of values by both patient and physician, as well as the feeling of being uninformed caused uncertainty in both parties [20].

It seems to be a challenging task to fully inform all patients. As described in the interviews, some patients seem to be unable to comprehend the information that is provided. This can be caused by cognitive impairment, fear or unwillingness to hear bad news. A study conducted in the Netherlands showed that patients with an incurable lung cancer “chose” to ignore bad news and showed a false optimism about recovery, not wanting to hear the bad news and only focus on treatment options [21]. A Belgian study advised to gradually deliver the message of a diagnosed incurable, terminal disease and prognosis [22]. Relatives of terminally ill patients told they were informed about the severity of the illness too late in the process (often within 1 month of the patient’s death). At the same time, relatives did not know to what extent they wanted to be informed, and expressed difficulty in comprehending and accepting the message that they were told [23]. In another study, among general practitioners (GP’s) and patients receiving palliative care, apart from physicians’ availability, the hesitation of both patients and GPs to talk about a bad prognosis was a main barrier to good communication [24].

Cultural or religious differences in background between the patient (or relatives) and the physician played an important role. Physicians described they felt unable to reach patients or relatives from cultures they were not familiar with, and felt that often the relatives had a different perception of treatment or death. This was more often described in cases where the physician wanted to stop treatment, against the wishes of the patient or relatives. Different religions were described, with the majority being a Muslim background, or a specific Christian background (for instance Jehovah’s witnesses). The physicians’ lack of knowledge of the cultural and social background of their patients can complicate shared decision making. Those experiences will for a great part be the basis of the end-of-life preferences of that same patient [25]. Understanding the patients’ explanatory models about illness, treatments and death can help make sense of seemingly unreasonable actions and decisions. The emphasis on patient autonomy and informed consent can clash with family-oriented cultures, where decisions are made by relatives. There is no reason to question requests from the appointed legal representatives, if a patient expressed the wish to let that person make decisions for them. Complying to the legal representative is a direct expression of following patient preferences.

Language differences can cause problems. This did not seem to be an important factor in the interviews, but can most certainly be of importance when communicating with patients. Translation by a relative may influence the content of the conversation, either due to misinterpretation of medical information, or driven by cultural beliefs that patients should be shielded from bad news. Taboo topics may be left out in translation. The use of trained medical interpreters should perhaps be standard of care when dealing with patients who do not speak the same language the physician does.

In conclusion, we found that physicians felt uncomfortable when there was disagreement between themselves and patients or relatives. Frustration was especially felt when relatives spoke on behalf of the patient, while there was no evidence the desired decision was ever expressed by the patient. The physicians were in doubt whether or not the desire for treatment was prompted by the previous wishes of the patient, or stemmed from the personal wish of the family not to lose a relative. Although this was comprehensible seen from the point of view of the family, the patients’ best interest was still the most important factor in the physicians’ decision to not start or stop a treatment. Differences in background, especially religious ones, were often mentioned as complicating communication. Although all physicians were trained in the Netherlands and now working within the same country, with its multicultural diversity, results cannot automatically be extrapolated to other countries or cultures. However, it is likely many of the experienced situations and frustrations are recognizable to physicians and patients in countries and cultures all over the world.

### Recommendations

Based on this qualitative research, impasses between physicians and patients are less likely to occur if the patient is well informed, capable of making treatment decisions and there is an existing relationship between patient and physician. Efforts must be made to establish a bond of trust between patient, relatives and physician. The use of advance directives should be encouraged. In case of an impasse between a physician and patient or relative, advice can be sought from other professionals. However, in the end it is still the physician who will have to decide whether or not a treatment is to be started, with the best interest of the patient at heart, even when this is not congruent with the wishes of the family.

### Strengths

To the best of our knowledge, this is the first study investigating the reaction of physicians on an impasse between them and a patient or relatives of that patient.

A wide variety of physicians was interviewed, from hospitals as well as general practitioners.

### Limitations

There are limitations to this study. First, participating physicians work in the same region in the Netherlands; therefore, the results cannot be extrapolated to other regions of the same country, other countries or different cultures. Second, in acquiring data from interviews, we express their narrative view of their experiences and perceptions.
